# Experimental and Numerical Analyses of Temperature-Reducing-Effect by Heat of Water Evaporation on a Moss-Greening Ceramic Utilizing Waste Silica

**DOI:** 10.3390/ma11091548

**Published:** 2018-08-28

**Authors:** Kentaro Yasui, Ayako Tanaka, Kenichi Ito, Minoru Fujisaki, Hiroyuki Kinoshita

**Affiliations:** 1 Department of Engineering, University of Miyazaki, 1-1 Gakuen-Kibanadai-Nishi, Miyazaki 889-2192, Japan; k-yasui@cc.miyazaki-u.ac.jp; 2 Graduate School of Engineering, University of Miyazaki, 1-1 Gakuen-Kibanadai-Nishi, Miyazaki 889-2192, Japan; hj13028@student.miyazaki-u.ac.jp; 3 Center for International Relations, University of Miyazaki, 1-1 Gakuen-Kibanadai-Nishi, Miyazaki 889-2192, Japan; itoken@cc.miyazaki-u.ac.jp; 4 Fuji Silysia Chemical Ltd., Hichiya Aza-Kihara 16303-3, Hyuga City, Miyazaki 883-0062, Japan; fujisaki@fuji-silysia.co.jp

**Keywords:** waste silica, moss-greening material, heat of water evaporation, radiant heat-reducing effect, FEM analysis

## Abstract

To recycle silica byproducts and to moderate the heat-island phenomenon, a porous ceramic was prepared by mixing waste silica powder with clay, and then firing the resultant mixture. By exploiting the high water-absorption capacity of the resulting ceramic, a greening material in which the porous ceramic was covered with moss was produced. The suppression effect of the temperature increase caused by solar-radiant heat on the moss-covered ceramic, was investigated quantitatively using the following procedure. First, the surface temperature change of the water-absorbing moss-covered sample during solar-radiant heat reception, and the amount of water that evaporated from the sample were measured simultaneously. Then, the heat of evaporation was estimated from measurements of the rate of water evaporation. Next, to investigate how much the sample temperature was reduced by heat of water evaporation, the temperature change of the sample when the heat of water evaporation was absorbed from the sample, was simulated by performing Finite Element Method (FEM) analysis. The summary of the results was as follows. (1) The primary factor of the temperature-reduction-effects on the moss-covered sample was action of heat of water evaporation. Therefore, the moss-covered sample did not exhibit much of the suppression ability of the temperature increase caused by solar-radiant heat, when the sample did not contain sufficient water. (2) This analytical method enabled us to simulate with a relatively high accuracy, the temperature change of a water-absorbing sample during solar-radiant-heat reception. Especially, the method enabled us to investigate visibly the influence of water evaporation-heat on the sample temperature, in addition to the influences of the emissivity of the sample, and the apparent specific heat and thermal conductivity changes due to water content in the sample.

## 1. Introduction

Silica is used in various products, including desiccants, toiletries, and filtration materials. However, many byproducts are produced during its manufacturing, and many of these are discarded as industrial waste. Effective use of waste silica is therefore desirable. Countermeasures to the urban heat-island phenomenon have also become increasingly important [1,2]. Achieving summer power savings is desirable.

A water-retention technique, and the greening of pavements and buildings are effective countermeasures to the urban heat-island phenomenon [3,4,5,6]. Particularly, rooftop greening of buildings is expected as the countermeasure because it can suppress the circumference temperature increase caused by solar-radiant heat, and a room-temperature reduction can also be achieved [7,8,9,10,11,12,13,14,15].

However, rooftop greening has not advanced significantly because it has many issues as described below.

(1) Rooftop greening is subject to weight limitations.
(2) Installing greening plants on rooftops is expensive.
(3) It is necessary to protect rooftops from corrosion or deterioration caused by greening plants.
(4) Greening-plant maintenance is labor-intensive.

Developing simple non-problematic greening materials is clearly desirable [16,17,18,19,20].

In consideration to that situation, we produced a porous ceramic by mixing waste silica powder with clay, and then firing the resulting mixture. By taking advantage of the high water-absorption capacity of the resulting ceramic, we produced a greening material in which the porous ceramic was covered with moss. The moss-covered ceramic is lightweight, and can be attached to rooftops without complicated adhesive processes because the ceramic base can be bonded to the floor. Moss maintenance is straight-forward. 

There are several studies on the suppression effect of the temperature increase caused by solar-radiant heat on the moss-greening materials [21,22]. Some of the studies have reported that moss-greening materials do not exhibit much temperature reducing ability, if they do not contain enough water [23,24]. It is believed to be because the sample’s apparent physical properties, such as specific heat and thermal conductivity, change due to water included in the sample [25], or the sample is deprived of the heat necessary for evaporating water [26,27,28,29]. The influence of moss transpiration may also be included in other factors. 

However, their reports generally seem to be phenomenological considerations, although they indicate that water included in the sample has a large effect on temperature reduction. It is unknown how much heat of water evaporation and moss transpiration, affect the sample temperature reduction. It is desirable for factors on temperature reduction effects of moss greening materials to be clarified quantitatively, for the optimal design [30]. It must be able to indicate clearly the suppressible temperature and duration on every moss-greening material, and its use must be encouraged. Therefore, this study aims to clarify quantitatively the suppression effect of the temperature increase caused by solar-radiant heat on moss-covered ceramic. Specifically, clarifying quantitatively the influence of heat of water evaporation on the temperature reduction-effect of the sample, is a primary target.

Currently, it seems that a method that can analyze quantitatively, the influence of heat of water evaporation on the temperature of the moss-greening materials has not been established [17,18], although some analyses have been performed [31,32,33,34]. The reason is thought to be because it is difficult to predict the temperature change of the sample, whilst the phase change phenomena of water is occurring. The research on moss-greening materials has been mostly conducted from the viewpoint of agriculture, botany, and environmental engineering thus far. It is considered that an approach from the viewpoint of engineering, based on thermodynamics, is effective for clarifying quantitatively, the temperature reduction effect by water evaporation heat on the moss-greening materials. Therefore, we used an analytical method as follows:

(1) The surface temperature change of the water-absorbing moss-covered sample during solar-radiant heat reception, and the amount of water that evaporated from the sample were measured simultaneously. Then, heat of evaporation was estimated from measurements of the rate of water evaporation. 

(2) To investigate how much the sample temperature was reduced by heat of water evaporation, the temperature change of the sample was simulated by FEM analysis, under the assumption that the heat of water evaporation was absorbed from the sample. 

In this paper, first, the overview of developed moss-covered ceramic was described. Next, the surface temperature change and the amount of water evaporation on the moss-covered sample during a constant radiant heat reception were measured in laboratory experiments, which were performed using a halogen lamp instead of solar radiation. 

Based on the experimental results, the suppression ability of the temperature increase caused by radiant heat on the moss-covered sample was first verified, and the influence of water content in the sample on the temperature was investigated. Especially, by employing how to irradiate the sample surface with a certain amount of radiant heat, the relationship between the quantity of radiation heat and the water evaporation rate of the sample, and the sample temperature change while water was evaporating from the sample were investigated in detail. Then, the temperature change of the sample was simulated by FEM analysis using data measured in the experiments. The influence of water evaporation heat on the sample temperature was investigated. 

Finally, field experiments and FEM analyses were performed, and the suppression ability of the temperature increase caused by solar-radiant heat on the moss-covered sample and the temperature reduction effect by the heat of water evaporation, were evaluated quantitatively. In the FEM analyses, the influences of specific heat and thermal conductivity changes caused by water included in the sample on the sample temperature, and the thermal emissivity of the sample were also investigated.

## 2. Materials and Methods

### 2.1. Materials

#### 2.1.1. Production of the Moss-Covered Ceramic

**Figure 1:**
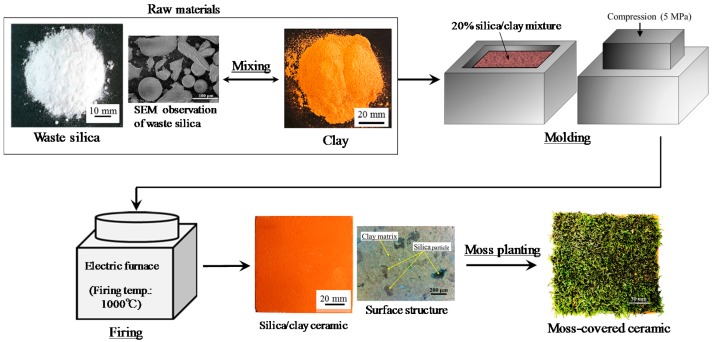
Process used to prepare the moss-greening material.

Figure 1 shows the process used to prepare the moss-covered ceramic material. Porous ceramic from clay and waste silica powder were covered with moss. Clay with chlorite as the major mineral (produced in Miyazaki, Japan [35]), and waste silica powder as a byproduct from merchandise manufacturing, were used to produce the porous ceramic base. Table 1 shows the chemical composition of the clay after firing and the waste silica powder. 

Silica powder (20% of total mass), with a maximum particle size of 0.2 mm was mixed with clay. The mixtures were solidified by pressing into molds at 5 MPa. The resulting samples were heated in an oxidizing atmosphere, at a heating rate of 100 °C·h^−1^, up to the firing temperature (1000 °C), in an electric furnace (KY-4N, Kyoei Electric Kilns Co., Ltd., Tajimi, Japan). The samples were held at the firing temperature for 1 h, and then left to cool to room temperature in the furnace. The moss-covered sample was produced by distributing moss (Racomitrium canescens), with a particle size of ~5–10 mm, at a density of 500 g per unit area, over the ceramic base, which had first been coated with an adhesive solution (Kuricoat, C710, Kurita Water Industries Ltd., Tokyo, Japan). The thickness of the moss was approximately 5 mm.

#### 2.1.2. Physical Properties of the Ceramics

**Figure 2:**
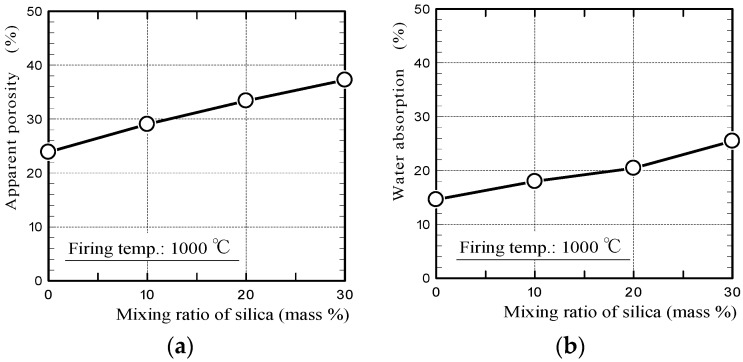
(**a**) Apparent porosities and (**b**) water absorption values of the ceramics.

**Figure 3:**
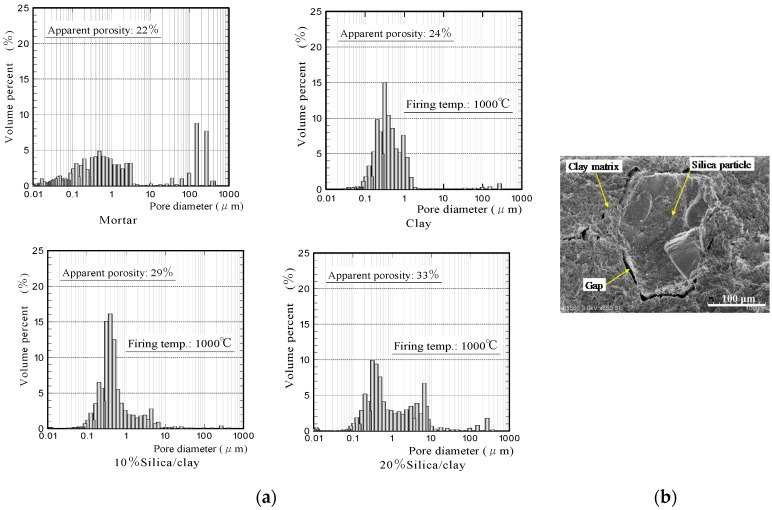
(**a**) Pore-diameter distributions of ceramics and (**b**) a SEM image of the surface structure of 20% silica/clay ceramic.

Figure 2 shows the apparent porosities and water absorption values of silica/clay ceramics. Figure 3 shows the pore-diameter distributions of the ceramics, and a scanning electron microscope (SEM) image of the surface structure of 20% silica/clay ceramic. The apparent porosities and pore-diameter distributions of the ceramics, were measured using a mercury porosimeter (Auto Pore IV 9500, Micromeritics Instrument Corporation, Norcross, GA, USA). The pore-diameter distributions of the ceramics are shown along with that of the mortar sample, which was prepared by mixing ordinary Portland cement with a fine aggregate in a 1-to-3 mass ratio. 

The porosities of the silica/clay ceramics increased when increasing the amount of silica powder in the silica/clay mixture. The ceramic pore size increased when silica powder was mixed with clay before firing, which increased the water-absorption capacity. From Figure 3b, it is considered that both silica particle and clay shrunk after firing, and gaps were generated between the silica particle and clay matrix. The porosity and water absorption on the mortar sample were approximately 22% and 5%, respectively. The values were much smaller than those of ceramic made from clay alone. 

**Figure 4:**
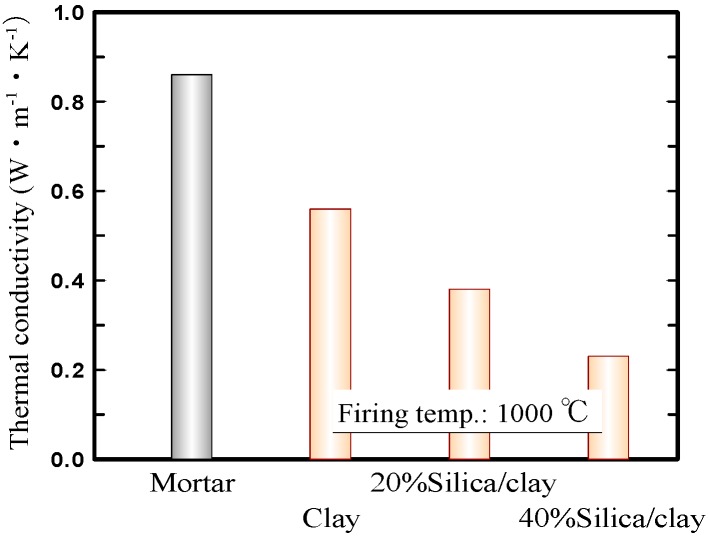
Thermal conductivities of the ceramics.

Figure 4 shows the thermal conductivities of the mortar, ceramic from clay alone, and silica/clay ceramic samples. Here, the thermal conductivities of ceramic samples were measured using a laser flash method (LFA457 MicroFlash, NETZSCH-Geratebau GmbH, Selb, Germany). That of the mortar sample was quoted from literature, according to Reference [36]. The graph exhibits that the thermal conductivities of the silica/clay ceramic samples were lower, than those of a ceramic sample from clay alone and a mortar sample. The silica/clay ceramics were expected to be capable of lowering indoor temperatures in the summer, when used as tiles on exterior walls and building rooftops.

**Figure 5:**
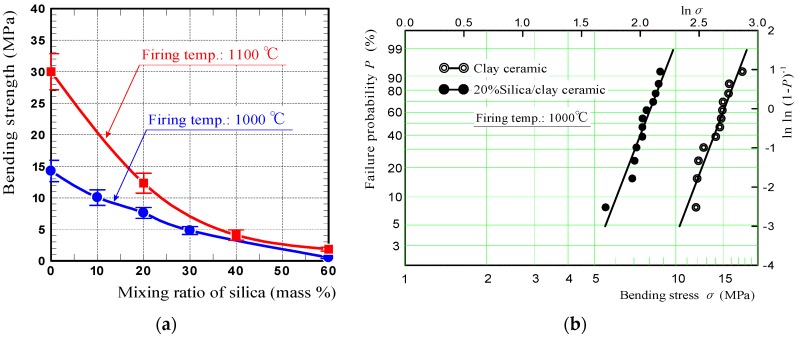
(**a**) The bending strengths of silica/clay ceramics and (**b**) Weibull plots of the bending strengths of 20% silica/clay and clay ceramics.

Figure 5a shows the bending strengths of silica/clay ceramics. These measurements were obtained by performing four-point bending tests. The data points are average bending strengths calculated from the measurements of 5–11 specimens, and error bars indicate standard deviations. In the graph, the bending strengths of silica/clay ceramics fired at 1100 °C, were shown along with those of the ceramics fired at 1000 °C. Figure 5b shows Weibull plots of the bending strengths of 20% silica/clay, and clay ceramics fired at 1000 °C. The *x*- and *y*-axes, express logarithms of the bending strength and specimen cumulative failure probability *P*, respectively. Although the silica/clay ceramic bending strength decreased with increasing mixing ratio of silica powder, because the ceramic porosity increased via increasing the amount of silica powder, 20% silica/clay ceramic fired at 1000 °C had sufficient strength to bond to the roof floors using an adhesive, and also met the 4 MPa strength criterion for vegetative pavement blocks.

### 2.2. Experimental Methods

#### 2.2.1. Laboratory Experiment

**Figure 6:**
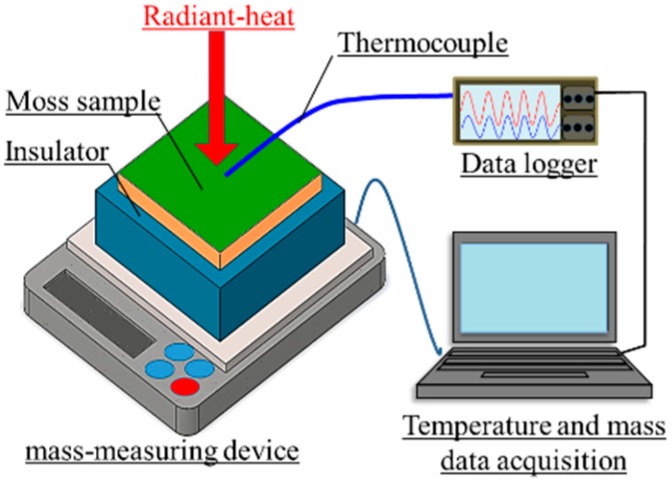
A schematic of the measurement setup in a laboratory experiment.

Figure 6 shows a schematic of the measurement setup in a laboratory experiment. The moss-covered sample surface was irradiated using a halogen lamp (PROmate, PHLS-500W, Daido Corporation, Tokyo, Japan) in a dark room. The front surface-temperature change of the sample was measured using a thermocouple (DG-K-5m-Y, AS ONE, Osaka, Japan). Simultaneously, the amount of water that evaporated from the sample was measured using a mass-measuring device (Electronic balance EK-6000i, A&D, Tokyo, Japan). Here, the insulator (JIS A9511, Styrofoam, DowKakoh, Tokyo, Japan [37]) with lengths of 150 × 150 mm, and a thickness of 50 mm, was put between the sample and mass-measuring device, so that the sample temperature was not affected by the temperature of the mass-measuring device. The intensity of radiation was approximately 900 W/m^2^, as measured using a pyranometer (LP PYRA02, Delta OHM, Padua, Italy).

Mortar and ceramic samples without moss in non-water-absorbing state, and the mortar sample without moss in the water-absorbing state were used in addition to the moss-covered sample in the water-absorbing state. Their samples were square-shaped specimens, with lengths of 100 × 100 mm, and a thickness of 10 mm. For the samples in the water-absorbing state, they were used for the experiments in an almost saturated-water absorbing state. The samples were prepared by being immersed in water, in the room from the previous day. By researching in detail, the amount of water evaporated from the sample and the rate of water evaporation for the duration of the temperature measurement, the influence of water content in the sample on the temperature was investigated. By comparing the surface-temperature change of the moss-covered sample, with that of the mortar sample, the suppressing ability of the temperature increase caused by radiant heat on the moss-covered sample was evaluated. 

#### 2.2.2. Field Experiment

**Figure 7:**
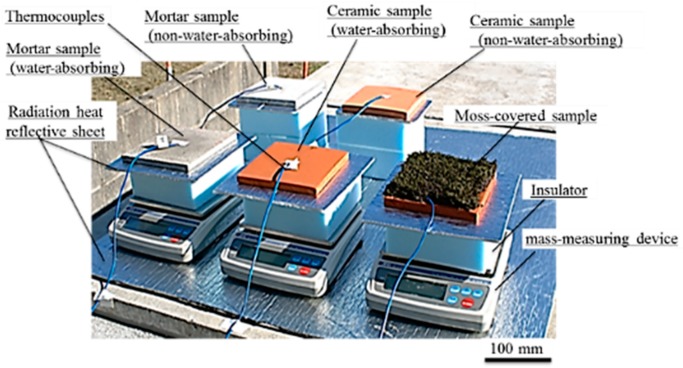
Measurement setup in field experiment.

A field experiment was performed outdoors on a hot day when the maximum air temperature was 30 °C or more. Figure 7 shows a photograph of the measurement setup in the field experiment. In this experiment, to increase the water-absorption capacity of the samples, a larger size of samples was used. The samples were square-shaped specimens, with lengths of 150 × 150 mm, and a thickness of 10 mm. In addition, the temperature of the insulator and mass-measuring device, considerably increased in the laboratory experiment, and in the measurement setup of the field experiment, an aluminum radiation heat reflective sheet with reflectivity of 95–97% (Alumi-shanetsu-sheet, AdHoc, Toyama, Japan) was inserted additionally, between the sample and insulator. The reflective sheet was also spread out in the surroundings of the mass-measuring device, to minimize the temperature increase caused by solar-radiant heat of the insulator and mass-measuring device. The reflective sheet inserted between the sample and insulator was a square-shaped sheet with lengths of 250 × 250 mm, and a thickness of 4 mm. The front surface-temperature changes of the samples and the amount of water that evaporated from the samples, were measured in the same manner as the laboratory experiment.

### 2.3. Methods of FEM Analyses

#### 2.3.1. Overview of the FEM Analyses

**Figure 8:**
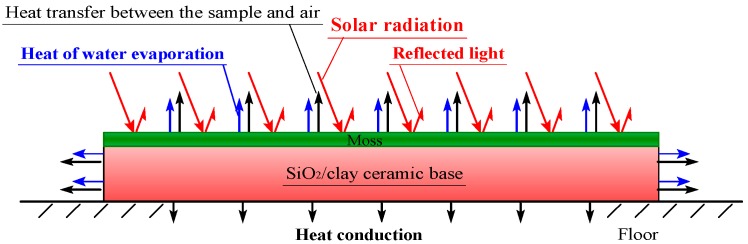
Schematic diagram of heat balance on the moss sample.

Figure 8 shows a schematic diagram of the energy balance on a moss-covered sample, during solar-radiant heat reception. The sample temperature increases by receiving the energy, in which the energy of the reflected light is removed from that of solar-radiation. In the front surface and sides of the sample, the heat transfer by convection, occurs between the sample and air. In the bottom of the sample, heat conduction occurs between the sample and floor. In addition, heat necessary for the water evaporation must be absorbed from the sample, through heat exchange when water included in the sample is evaporated. It is conjectured that there is also energy consumption by photosynthesis and transpiration of moss. Here, we defined that “transpiration” is a phenomenon in which water moves to the atmosphere through vegetation, and “evaporation” is a phenomenon in which water simply vaporizes.

As for the thermodynamic factors, regarding the suppression effects of temperature increase caused by solar-radiant heat, they are heat quantity which the moss-covered sample receives from solar-radiation, heat quantity absorbed from the sample due to water evaporation, and changes of material properties, such as specific heat and thermal conductivity, due to water content in the sample. Although there is also energy consumption by photosynthesis and transpiration of moss, it is very difficult to investigate them thermodynamically at present. 

The ratio of the heat quantity, which the moss-covered sample receives from solar-radiation, corresponds to an emissivity of the sample. To clarify these influential factors as much as possible, we investigated the temperature change of the water-absorbing moss-covered sample during solar-radiant heat reception in the following procedure.

(1) The temperature changes of mortar and ceramic samples in a non-water absorbing state were simulated by FEM analyses, under the assumption that their samples possessed an identical emissivity [38]. The heat quantity the sample received from solar-radiation, was calculated by multiplying the solar-radiant heat measured in the experiment by the sample emissivity. 

(2) The heat quantity absorbed from the sample through heat exchange due to water evaporation, was estimated from values of the rate of water evaporation measured in the experiment. 

(3) The temperature changes of the water-absorbing samples were simulated, under the assumption that the estimated water evaporation-heat was absorbed from the sample.

#### 2.3.2. FEM Model and Computational Conditions

**Figure 9:**
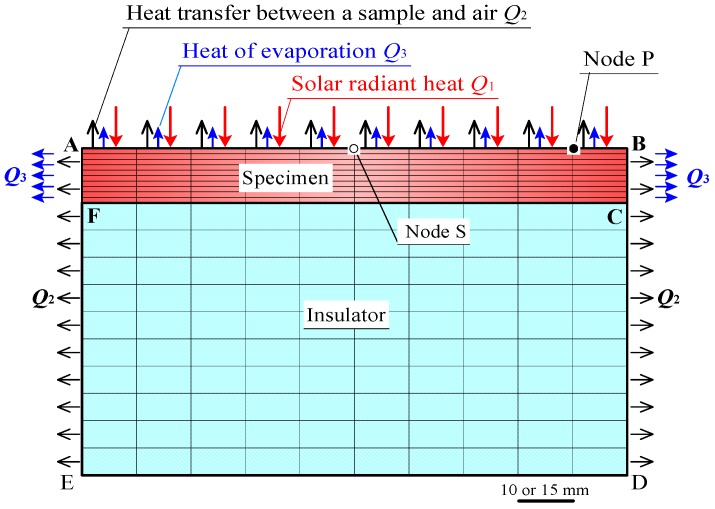
FEM model.

Figure 9 shows the two-dimensional FEM model. A Marc/Mentat FEM code was used. The FEM model corresponded to the cross section of a specimen and an insulator, which were used in the experiments. Lines AB and CF, represent front and rear sample surfaces, respectively. The lengths of AB, CF, and DE in the FEM model were 100 mm, in case of the laboratory experiments, and were 150 mm in field experiments. A uniform heat flux *Q*_1_ by radiant heat on AB, and a uniform heat flux *Q*_2_ by heat transfer between the sample and air, were distributed on AB, BD, and EA, except for the insulator bottom DE. A heat flux *Q*_3_, which was absorbed from the sample due to water evaporation, was distributed on AB, BC, and FA. The heat flux *Q*_3_ was obtained as follows:

(1) The water evaporation heat per mass was 2452–2380 kJ·kg^−1^ (20–50 °C), as in Reference [36].
(2) The quantity of heat did not change significantly in this temperature range. Therefore, the heat of evaporation per unit mass was approximated to a constant value of 2400 kJ·kg^−1^. The heat of water evaporation per unit time, was estimated by multiplying the rate of water evaporation for each sample by the heat of water evaporation per unit mass.
(3) The heat flux *Q*_3_ was approximated by dividing the heat of water evaporation per unit time absorbed from each sample, by the sample surface areas of AB, BC, and FA.

Table 2 shows the computational conditions. The values of specific heat on the mortar sample, and the specific heat and thermal conductivity on the insulator were quoted from literatures, respectively [37]. Values of the sample emissivity were also quoted from literature [38], and it was 0.9. The coefficient of heat transfer between the sample and air was determined by trial-and-error, so that the surface temperature changes of the mortar and ceramic samples in a non-water absorbing state, could match with those in experiments as much as possible.

## 3. Results and Discussions

### 3.1. Laboratory Experiment

#### 3.1.1. Experimental Results

**Figure 10:**
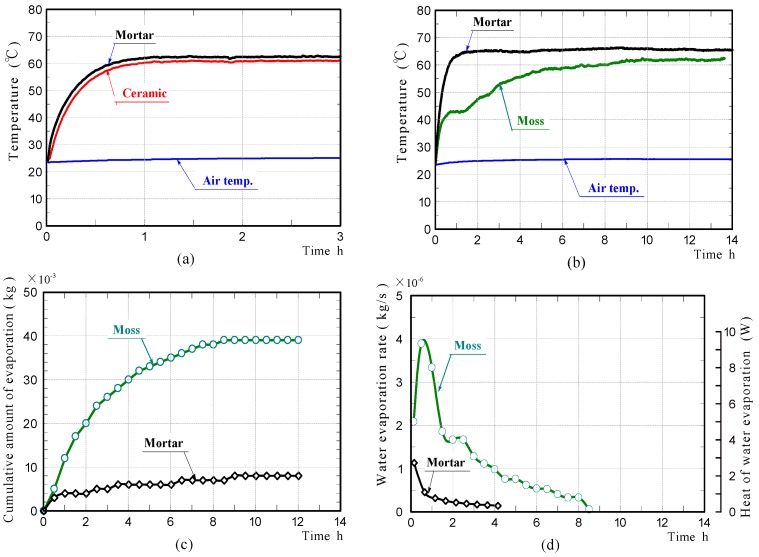
Surface-temperature changes of the mortar and ceramic samples in the non-water absorbing state (**a**), the mortar and moss-covered samples in the water absorbing state (**b**), amounts of water evaporated from the samples (**c**), and water evaporation rate (**d**) in laboratory experiments.

Figure 10a,b shows the front surface-temperature changes of the ceramic and mortar samples without moss in the non-water absorbing state, and those of the moss-covered and mortar samples in the water-absorbing state, during halogen lamp irradiation. The front surface-temperatures of the mortar and ceramic samples in the non-water absorbing state, were comparable at all times. Their temperatures increased rapidly during the initial 1.0 h of irradiation, then became almost constant. The front surface-temperature change of the mortar sample in the water-absorbing state was almost the same as that in the non-water absorbing state. In contrast, the front surface-temperature of the moss-covered sample increased rapidly during the initial 0.5 h of irradiation, then plateaued at a low temperature, for the next approximately 1.0 h. The front surface-temperature then increased at a moderate rate with further irradiation time, eventually becoming almost constant and slightly lower than the final temperature of the mortar sample. These results exhibited that the moss-covered sample in the water-absorbing state, could suppress the temperature increase caused by radiant heat, better than the mortar sample.

Figure 10c,d shows the cumulative water that evaporated from the moss-covered and mortar samples, and the rates of water evaporation. For the moss-covered sample, a considerable amount of water continued to evaporate during approximately 9 h, due to its high water-absorption capacity. For the mortar sample, a slight amount of water evaporated from the sample only during the initial approximately 4.0 h of irradiation. The results of the laboratory experiments are summarized as follows.

(1) While the moss-covered sample contained sufficient water, or water was evaporating from the sample, the surface-temperature of the sample was lower than that of the mortar sample.
(2) The surface-temperature of the moss-covered sample that contained slight amounts of water, went up to nearly the temperature of the mortar sample that contained slight amounts of water. The difference in their final temperatures was only 2~3 °C.
(3) The above results confirmed that the moss-covered sample does not exhibit much temperature-reducing ability, when the sample does not contain sufficient water, and that water included in the sample significantly affects the temperature reduction. This leads to the conclusion that, it is desirable to use the moss-covered sample in a sufficient water-absorbing state.

#### 3.1.2. The Results of FEM Analyses

**Figure 11:**
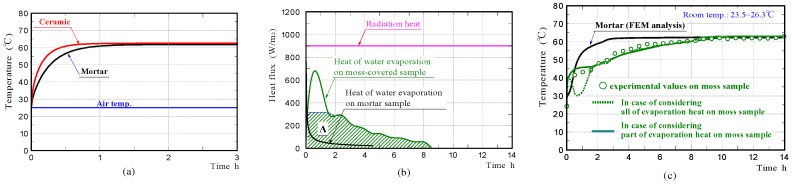
Surface-temperature changes of the mortar and ceramic samples in the non-water absorbing state obtained by FEM analyses (**a**), heat of water evaporation (**b**), and surface-temperature changes of the mortar and moss-covered samples in the water absorbing state (**c**) obtained by experiments and FEM analyses.

Figure 11a shows the front surface-temperature changes on the mortar and ceramic samples in the non-water absorbing state, whilst the samples were subjected to constant radiation (900 W/m^2^). The graphs exhibited temperature changes at Node P (see FEM model in Figure 9), with increasing time. The results confirmed that the surfaces of the mortar and ceramic samples in the non-water absorbing state, had comparable temperatures at all times like those in the experiments. It is conjectured that there was no significant difference in the emissivity of the mortar sample and ceramic sample. 

Figure 11b shows the heat of water evaporation on the moss-covered and mortar samples, which were estimated from the values of the rates of water evaporation measured in the experiments. The heat quantity of water evaporation is shown, along with the levels of intensity of radiation. Here, the heat quantity of water evaporation was obtained by dividing the heat of water evaporation per unit time by the sample surface areas, except for the bottom. For the region which is hatched, it is described later.

Figure 11c shows the front surface-temperature changes of the mortar and moss-covered samples in the water-absorbing state, which were simulated by FEM analyses, under the assumption that all the heat quantity of water evaporation was absorbed from only the sample through heat exchange. The curve drawn by the green dotted line, represents the front surface-temperature change on the moss-covered sample. Regarding the green solid line, it is described later. The surface temperature of the moss-covered sample, disagreed with that measured experimentally during the initial approximately 1.5 h of irradiation, although it agrees well with that afterward. 

We investigated the phenomenon that the surface temperature of the moss-covered sample plateaued at a nearly-constant low temperature, temporarily in the early stage. A phenomenon like this has also been observed in the temperature change of a different type of porous ceramic in the water-absorbing state, so far [8]. The surface-temperature of the moss-covered sample was kept at a nearly-constant temperature, despite the heat of water evaporation changing continuously, for the duration of the measurement. The heat quantity absorbed from the sample due to water evaporation, during the initial approximately 1.5 h, was clearly smaller than that estimated from the measurement of the rate of water evaporation. Therefore, it is guessed that only part of the water evaporation-heat was absorbed from the moss-covered sample, and the remaining water evaporation-heat was absorbed from the surrounding substances, such as an insulator, a mass-measuring device, and sample peripheral air. Particularly, it is conjectured that the heat necessary for water evaporation was exchanged with sample peripheral air, so that the sample temperature was kept at a nearly-constant temperature.

In order to verify the above assumption, we simulated the temperature change of the moss-covered sample by accounting for only part of the water-evaporation-heat. In the graph on the heat of evaporation shown in Figure 11b, the heat quantity shown in area A, which is hatched, was used for FEM analysis. The maximum heat quantity was limited to the heat quantity at the time of completion, when the sample surface was kept at a nearly-constant temperature. The green solid line in the graphs shown in Figure 11c, shows the surface-temperature change of the moss-covered sample. The surface temperature of the moss-covered sample agreed well with that measured experimentally. From the result, it was considered that the heat quantity, which excluded the heat quantity shown in area A, from the whole heat quantity of water evaporation during the initial 1.5 h of irradiation, was absorbed from the sample surroundings.

**Figure 12:**
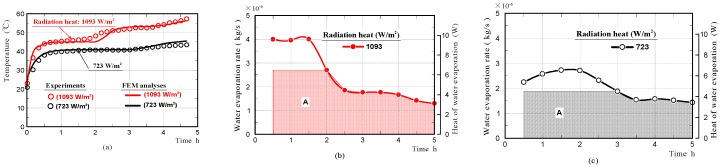
Surface-temperature changes of moss-covered samples obtained by experiments and FEM analyses while the sample was subjected to radiation of 723 or 1093 W/m^2^ (**a**), and water evaporation rates and heat of water evaporation (**b**,**c**).

Furthermore, we investigated whether the phenomenon that the surface temperature of the moss-covered sample was kept at a nearly-constant low temperature temporally, also occurred when the sample was subjected to different amounts of radiation. Figure 12a shows the surface-temperature changes on the moss-covered sample, whilst the sample was subjected to radiation of 723 and 1093 W/m^2^, respectively. Here, the intensity of radiation was changed by changing the distance between the halogen lamp and the front surface of the moss-covered sample. The results confirmed that the surface temperature of the moss-covered sample in the water-absorbing state plateaued, at a nearly-constant low temperature temporally when the sample was also subjected to different amounts of radiation. The nearly-constant temperature became higher with an increase in radiation, and the duration when the surface-temperature was kept at a nearly-constant temperature decreased with an increase in radiation. 

Figure 12b,c shows the rates of water evaporation and the heat of evaporation on the moss-covered sample. We also simulated the temperature change of the moss-covered sample using not the whole heat quantity of evaporation, but just one part of the heat quantity shown in area A. The red and black solid lines in the graphs shown in Figure 12a, show the front surface-temperature changes of the samples, which were simulated by FEM analyses. The front surface-temperature changes agreed well with those measured experimentally. The results supported that just one part of the heat quantity necessary for water evaporation was absorbed from the moss-covered sample, and the remaining heat quantity was absorbed from the surrounding substances, such as an insulator, a mass-measuring device, and air in the vicinity of the sample. 

**Figure 13:**
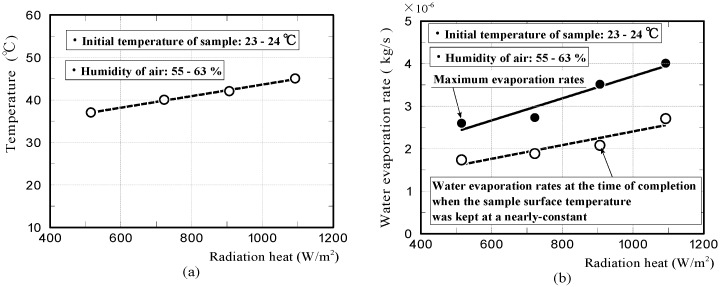
The relationship between the temperature of when the moss-covered sample surface was temporarily kept at a nearly-constant low temperature, and the quantity of radiation heat (**a**), the relationships between the maximum water evaporation rate of the sample and the quantity of radiation heat, and between the water evaporation rate at the time of completion when the sample surface was kept at a nearly-constant temperature and the quantity of radiation heat (**b**).

Figure 13a represents the relationship between the temperature of when the moss-covered sample surface was temporarily kept at a nearly-constant low temperature, and the quantity of radiation heat. It is considered that the graph exhibits a lower limit of the temperature that the moss-covered sample could lower, under this experimental condition. The solid and dotted lines shown in Figure 13b, represent the relationships between the maximum water evaporation rate of the moss-covered sample and the quantity of radiation heat; and between the water evaporation rate at the time of completion, when the sample surface was temporarily kept at a nearly-constant low temperature, and the quantity of radiation heat, respectively. The maximum water evaporation rate of the sample was generally, in proportion to the quantity of radiation heat. For the dotted line, it was considered that the graph exhibited a water evaporation quantity per unit time necessary to lower the sample temperature down to a nearly-constant low temperature. Here, the above water evaporation rate changes, depending on atmospheric temperature, humidity, and air velocity [39,40].

The above results indicated that, the temperature-reduction effect by the heat of water evaporation has a limitation. The suppressible temperature is up to the temperature of the sample peripheral air at most, even if a large amount of water evaporated from the sample. The sample temperature cannot understandably be reduced to the atmosphere temperature below, by the heat of water evaporation. For the rate of water evaporation, the sample with a high evaporation rate is not necessarily better. It is considered that the sample with a high water capacity, and capable of continuing to evaporate a certain extent amount of water for a long time is desirable.

### 3.2. Field Experiment

#### 3.2.1. Results of Field Experiments

**Figure 14:**
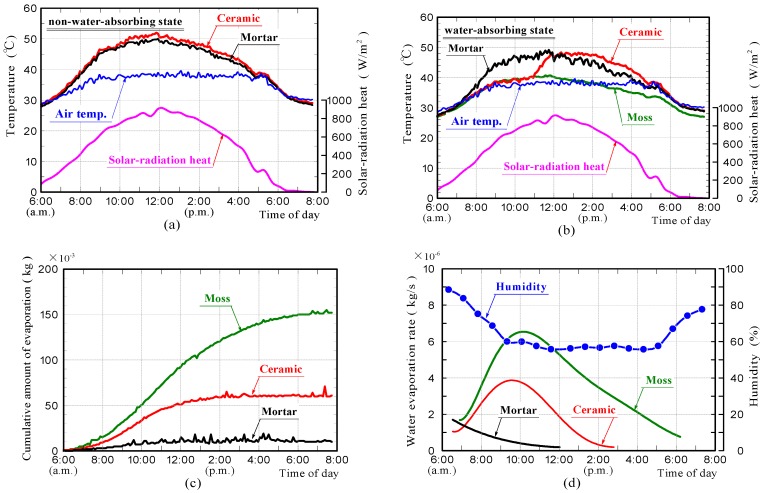
Surface-temperature changes of samples in the non-water absorbing and water absorbing states (**a**,**b**), amount of water evaporated from samples (**c**), and water evaporation rate and humidity (**d**) in field experiments.

Figure 14a–d shows the surface-temperature changes of samples in the non-water absorbing and water absorbing states, the cumulative water that evaporated from the samples and the rates of water evaporation in the field experiments, respectively. The weather was mostly sunny. It was a very hot day. The front surface-temperatures of the ceramic and mortar samples without moss in the non-water absorbing state, were comparable at all times, reaching a maximum of approximately 12 °C above air temperature. The surface-temperature of the moss-covered sample, was comparable to the air temperature. The surface temperature of the ceramic sample in the water absorbing state, was comparable to that of the moss-covered sample within approximately 5 h from the beginning of the measurement, after which the surface-temperature increased rapidly with further increases in time and became comparable to that of the ceramic sample in the non-water absorbing state. The surface temperature of the mortar sample in the water absorbing state was slightly lower than that of the mortar sample in the non-water absorbing state. 

Water evaporation from the moss-covered sample, occurred continuously throughout the durations of the measurement. The rate of water evaporation depended on the amount of solar-radiation, and was considerably larger than those of the other samples. Water evaporation from the mortar sample occurred only within approximately 6 h. Water evaporation from the ceramic sample, occurred within approximately 9 h. The results of the field experiments are summarized as follows.

(1) The temperature measurement results demonstrated that the moss-covered sample in the water absorbing state, could suppress the temperature increase caused by solar-radiant heat over the entire day, and that the ceramic sample in the water absorbing state could do it only for a certain period due to the smaller water-absorption capacity.

(2) The front surface-temperature of the ceramic sample in the water absorbing state, increased rapidly when the water evaporation was almost complete. 

#### 3.2.2. FEM Analyses on the Field Experiments

**Figure 15:**
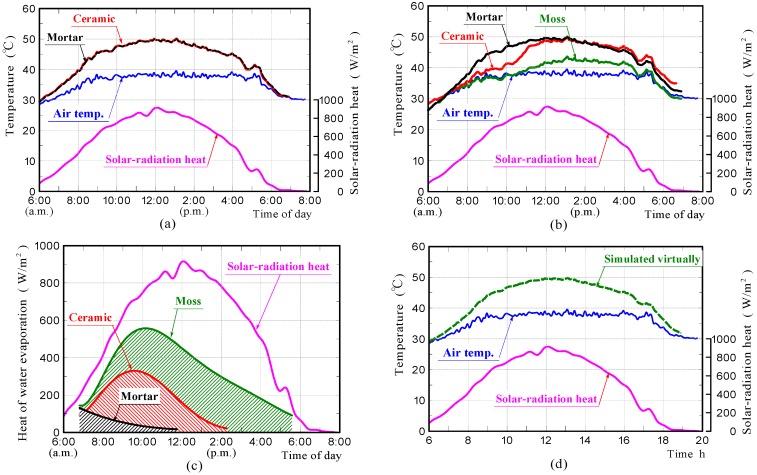
Surface-temperature changes of samples in the non-water absorbing and water absorbing states obtained by FEM analyses (**a**,**b**), heat of water evaporation (**c**), and surface-temperature changes of the moss-covered sample, which was simulated virtually using a different specific heat and thermal conductivity (**d**).

Figure 15a–c shows the surface-temperature changes of samples in the non-water-absorbing and water-absorbing states, which were simulated by FEM analyses, and the heat of evaporation estimated from values of the rates of water evaporation measured in the experiments, respectively. The surface-temperature changes of the samples in the water-absorbing state, which are shown in Figure 15b, were simulated under the assumption that all the water evaporation-heat shown in Figure 15c, was absorbed from the sample through heat exchange. In the case of the moss-covered sample, the heat of evaporation estimated from the values of the rate of water evaporation measured in the experiment was roughly half of the solar-radiant heat. In case of the water-absorbing ceramic sample, it was approximately one third of the solar-radiant heat. It is presumed that the quantity of heat absorbed from the sample due to water evaporation was very large.

The surface-temperature changes of samples in the non-water-absorbing state, which is shown in Figure 15a, agreed well with those measured experimentally. The results confirmed that the front surfaces of the ceramic and mortar samples without moss in the non-water absorbing state, had almost comparable temperatures during solar-radiant heat reception. It confirms that the ceramic and mortar samples possessed a comparable emissivity. 

Regarding samples in the water-absorbing state, which is shown in Figure 15b, the surface-temperature change of the mortar sample also agreed well with that measured experimentally. The result exhibited that the surface-temperature becomes almost comparable to that of the mortar sample in the non-water absorbing state, because heat of water evaporation was very small. 

The surface-temperature changes of the moss-covered and ceramic samples in the water-absorbing state, generally agree with those measured experimentally. The surface-temperature of the moss-covered sample concurs closely with that measured experimentally when assuming that all of the water evaporation-heat shown in Figure 15c, was absorbed from the sample, although the temperature was a little higher in the latter half of the measurement. Therefore, it was guessed that the heat of water evaporation significantly reduced the surface-temperature of the sample. 

As the reason why, the surface temperature of the moss-covered sample was lower than other samples, the apparent specific heat and thermal conductivity changes due to water absorption in the sample were also thought to contribute. We simulated the sample temperature change virtually, when assuming that the specific heat and conductivity of the moss-covered samples were 4180 J·kg^−1^·K^−1^ and 0.65 W·m^−1^·K^−1^ referring to the water physical properties, respectively. 

Figure 15d shows the front surface-temperature of the sample, which was simulated virtually, using the values of the above physical properties. It was found that the surface-temperature of the sample did not significantly change depending on the physical properties. It is believed that the primary factor of the temperature-reduction-effects on the moss-covered sample, was the action of heat of water evaporation. For the reason that the surface-temperature of the moss-covered sample was a little higher than that measured experimentally in the latter half of the measurement, the consumption of energy by photosynthesis and the influence of moss thickness etc. are assumed; but it is unknown at present. This is a future task.

The surface-temperature change of the ceramic sample in the water-absorbing state, also generally agrees with that measured experimentally. The result was capable of exhibiting clearly the phenomenon that the front surface-temperature of the sample increased rapidly from the middle of the measurement, and the temperature became comparable to that of the ceramic sample in the non-water absorbing state. This indicated that the reason for the front surface temperature of the ceramic sample in the water absorbing state increasing rapidly from the middle of the measurement, was because the heat of evaporation was no longer absorbed from the sample. 

## 4. Conclusions

To recycle silica byproducts, as well as to moderate the heat-island phenomenon, a porous ceramic with high water-absorption capacity was prepared by mixing waste silica powder with clay, and then firing the resultant mixture. Then a greening material in which the porous ceramic was covered with moss was produced. The suppression effect of the temperature increase caused by solar-radiant heat on the moss-covered ceramic was investigated. Specifically, the influence of heat of water evaporation on the temperature reduction-effect of the sample was investigated quantitatively. The summary of the results is as follows.

(1) The moss-covered sample did not exhibit much of the suppression ability of the temperature increase caused by solar-radiant heat, when the sample did not contain sufficient water. The primary factor of the temperature-reduction-effects on the moss-covered sample, was action of heat of water evaporation. 

(2) It was found that the moss-covered and ceramic samples in the water-absorbing state could suppress the temperature increase caused by solar-radiant heat, and the moss-covered sample could suppress the temperature increase for a longer time, compared with the ceramic sample due to the moss-covered sample possessing a higher water-absorption capacity. 

(3) Firstly, the rate of water evaporation on the water-absorbing sample was measured in the experiment. Next, heat of evaporation was estimated from values of the rate of water evaporation. Then, the temperature change of the sample was simulated by performing FEM analysis, under the assumption that the estimated evaporation-heat was absorbed from the sample. This method enabled us to simulate with a relatively high accuracy, the temperature change of a water-absorbing sample during solar-radiant-heat reception. Especially, it enabled us to investigate visibly, the influence of water evaporation-heat on the sample temperature, in addition to the influences of the emissivity of the sample, and the apparent specific heat and thermal conductivity changes caused by water absorption in the sample.

## Figures and Tables

**Table 1:** Compositions of inorganic substances in the clay and waste silica powder.

Component	Clay (Mass %)	Waste Silica (Mass %)
SiO_2_	64.3	93.7
Al_2_O_3_	23.0	5.57
Fe_2_O_3_	5.75	0.14
K_2_O	3.94	<0.1
MgO	1.63	<0.1
CaO	0.26	<0.1
TiO_2_	0.90	<0.1
SO_3_	0.12	0.54
others	<0.1	<0.1

**Table 2:** Computational conditions.

Parameters	Mortar	Ceramic		Insulator
Density (kg·m^−3^)	2238	1800		30
Specific heat (J·kg^−1^·K^−1^)	900	620		1000
Thermal conductivity (W·m^−1^·K^−1^)	0.86	0.38		0.03
Solar radiation (W·m^−2^)	measured values			
Air temperature (K)	measured values			
Thermal emissivity	0.9			
Coefficient of heat transfer between a sample and air (W·m^−2^·K^−1^)	Laboratory experiment		Field experiment	
	18		70	
